# Characterizing palliative care needs in people with or at risk of developing diabetic foot ulcers

**DOI:** 10.1177/20420188221136770

**Published:** 2022-11-14

**Authors:** Liliane Mendonça, Bárbara Antunes, Joana Rigor, Daniela Martins-Mendes, Matilde Monteiro-Soares

**Affiliations:** MEDCIDS – Departamento de Medicina da Comunidade Informação e Decisão em Saúde, Faculty of Medicine, University of Porto, Portugal; Primary Care Unit, Department of Public Health and Primary Care, University of Cambridge, Cambridge, UK; Centro Hospitalar de Vila Nova de Gaia/Espinho, EPE, Vila Nova de Gaia, Portugal; Departamento de Biomedicina, Faculty of Medicine, University of Porto, Porto, Portugal; MEDCIDS – Departamento de Medicina da Comunidade Informação e Decisão em Saúde, Faculty of Medicine, University of Porto, Rua Dr. Plácido da Costa, s/n, Porto 4200-450, Portugal; RISE@CINTESIS, Faculty of Medicine, University of Porto, Portugal

**Keywords:** cohort analysis, chronic illness, diabetes, diabetic foot, Palliative care, wound care

## Abstract

**Aims:**

Diabetic foot ulcers (DFUs) have a significant impact on a patient’s quality of life and life expectancy, with mortality rates comparable with malignant diseases. However, there is a lack of data regarding palliative care needs in this population. We aimed to characterize palliative care needs in people under diabetic foot surveillance using the Integrated Palliative care Outcome Scale (IPOS) and EuroQol-5D three-level version (EQ-5D-3L) and to assess differences between those with and without a DFU.

**Methods:**

We conducted a cross-sectional study with consecutive sampling inclusion of patients followed in a tertiary hospital’s Diabetic Foot Clinic between February and October 2019 with (*n* = 20) and without (*n* = 42) active DFU.

**Results:**

The most frequent symptoms encountered were pain, weakness or lack of energy, sore or dry mouth and drowsiness. Patients with an active DFU were significantly more likely to report feeling anxious or worried in comparison with those without (95% *versus* 55%, *p* = 0.002). Only 10% of the participants with an active DFU said that they were always able to share how they felt with family and friends as much as they wanted in comparison with 45% of those without (*p* = 0.006).

**Conclusion:**

Our study identified palliative care needs in patients under diabetic foot surveillance with and without DFU, including a significant presence of physical symptoms. Patients in both groups showed signs of emotional/psychological distress, with a higher manifestation in patients with DFU. To the best of our knowledge, this is the first study addressing and characterizing palliative care needs in this population.

## Introduction

Diabetic foot ulcers (DFUs) have a strong impact on patients’ quality of life, affecting their mobility, physical functioning, pain level, social activities and relationships.^1–3^ Patients with DFU present a greater impairment of health-related quality of life (HRQoL) when compared not only with the general population (in physical and mental HRQoL domains) but also with people with diabetes (in all domains).^4^

Once a DFU has developed, there is a greater chance of recurrence in the short term.^5^ Current literature estimates the recurrence rate to be around 22% per person-year, although recurrence rates vary widely in different regions.^6^ In fact, some authors advocate for the use of the term ‘in remission’ for the period that the patient is ulcer-free.^7^

It has also been shown that having a DFU also diminishes life expectancy, mainly due to cardiovascular events.^8^ This higher risk of death remained significantly so in those with a previous DFU even when adjusted for age, sex, visual or physical limitation, diabetes duration, number of comorbidities and previous lower extremity amputation (LEA).^9^ In an Australian study, people with DFU had a 5-year-mortality rate around 25% and a 10-year-mortality rate of 45%.^10^ This mortality rate is comparable with those reported for people with malignant disease as it estimated a 5-year pooled mortality for all reported cancers around 31%.^11^

Palliative care aims to relieve pain and other distressful symptoms and enhance patients’ quality of life in physical, social, psychological, cultural and spiritual domains.^12^ It is considered that this type of care should be provided to all people considered to be at the end of their lives. However, although most research has been conducted in the cancer setting, these patients represent only one-third of those needing palliative care.^13^

There is a paucity of studies on palliative populations with wounds,^14,15^ but it is acknowledged that palliative care should be integrated into wound care and be based on need and not on diagnosis.^16–20^

There is a small number of recommendations for integrating palliative and end-of-life care into the usual management of people with diabetes-related complications, although it is believed that people with diabetes and non-healing wounds would benefit from this approach.^21^

According to the Global Atlas of Palliative Care, suffering is health-related when it is associated with illness or injury, being moderate or severe when it cannot be relieved without the intervention of a healthcare professional, and when it compromises physical, social or emotional functioning.^13^ We believe that the diabetic foot context includes all these criteria and so should be further explored. (1) This opinion is based on the fact that having a diabetes-related foot wound has proven to be a risk factor for premature myocardial infarction and stroke^8^; (2) people with diabetes usually have other comorbidities, lose function, deteriorate slowly and also heal wounds slowly^17^ and (3) tend to present some degree of pain or discomfort, anxiety/worry, dementia (due to their age and to poor glycaemic control), depressed mood, diarrhoea or constipation (due to changes in the autonomous nervous system), itching (due to peripheral neuropathy and dry skin), weakness and wounds – being all of these symptoms accounted for in this Atlas.^13^

Patient-centred outcome measures (PCOMs) used systematically in clinical practice with immediate feedback to clinicians, though time- and resource-consuming,^22^ have been shown to improve the processes of care.^23^ This includes more discussions regarding the quality of life, better symptom recognition and increased referrals to palliative care services, which has been linked to an improvement in both emotional and psychological patient outcomes.^24^

The Integrated Palliative care Outcome Scale (IPOS) is a PCOM used to identify palliative needs in people with advanced diseases.^25^

For all this, we aimed to characterize palliative care needs in people with diabetes under diabetic foot surveillance in a tertiary care facility using IPOS and to compare differences between those with and without an active DFU.

## Methods

### Type of study and selection of participants

This was a cross-sectional study, with consecutive sampling. Inclusion criteria were patients followed in a Hospital Diabetic Foot Clinic between February and October 2019 who gave their oral and written consent to participate and were able to understand and respond to the questionnaires (ascertained by the research team). Patients were excluded if they presented active ulcers that were caused by other conditions besides diabetes, with malignant conditions or were bedridden and if there were difficulties in communication (such as cognitive impairment, hearing loss or inability to speak Portuguese). Each patient was included only once.

Patients without active ulcers are followed in our clinic if they are at risk of developing one based on the International Working Group on Diabetic Foot (IWGDF) classification. Therefore, they should have peripheral neuropathy with or without peripheral arterial disease, foot deformity or previous foot complications.^26^ However, we also receive referral requests from primary care or other departments from our hospital to assess patients with acute complaints or requiring podiatric care.^27^

### Data collection methods

Demographic (age, sex and educational level) and clinical data (type and duration of diabetes, treatment, diabetes-related complications, glycaemic control, physical autonomy, body mass index, waist circumference and foot characteristics) were collected by a podiatrist with more than 15 years of experience in Diabetic Foot.

Educational level was defined as up to *versus* higher than elementary school level, having in consideration that in 2010 still 20% of the population in Portugal aged 65 years and older were considered illiterate.^28^

We have considered as diabetes-related complications both micro and macrovascular complications, such as retinopathy, neuropathy, nephropathy, cardiovascular disease and peripheral arterial disease. The presence of each complication was asked to the patient during the interview and confirmed through medical record consultation.

Glycaemic control was assessed by the value of a glycated haemoglobin with less than 3 months registered in the patient electronic health record.

Physical impairment was defined as reported or observed difficulty in the patient to reach her or his own feet.^29^

In those with an active DFU, Site, Ischaemia, Neuropathy, Bacterial Infection and Depth (SINBAD) classification was used to describe them.^30,31^ This classification consists of identifying the presence of these six ulcer-related clinical features. Each component can be graded with zero (for absence) or one (for presence) and the total score ranges between zero and six.

Ischaemia was considered present when two or fewer pedal pulses were palpable in both feet, out of the possible four.^32^ Neuropathy was diagnosed using the Semmes-Weinstein monofilament and the tuning fork. When sensation was altered with at least one of the instrument, neuropathy was considered present.^33^ The definition of foot deformity followed the IWGDF recommendations.^32^ Infection was evaluated through clinical manifestations and using the Infectious Diseases Society of America (IDSA)/IWGDF classification.^31^ The area was calculated using the elliptical wound measurement and depth through visual inspection and by the use of a sterile probe, whenever necessary.

Afterwards, a clinical psychologist applied the Portuguese versions of the IPOS^34^ and EuroQol-5D^35^ questionnaires, in a quiet and private room made available at the Hospital.

The IPOS questionnaire is an instrument developed to measure palliative care needs that aim to generate a score for each item and an overall score.^25^ This tool is composed of 10 brief and easy to answer questions that address all the dimensions of an individuals’ life and has a free text section to identify the needs that have not been contemplated by the existing items. The items are on a 5-point Likert-type scale. Items were converted in three 0–100 scales: physical symptoms, emotional symptoms and communications/practical issues (higher scores correspond to higher severity symptoms/functionality). The Portuguese version has a good internal consistency (Cronbach’s α of 0.66) and very good reliability (> 0.80).^34^

The EuroQol-5D three-level version (EQ-5D-3L)^35^ is a widely validated and used generic instrument to measure HRQoL that allows producing utility values based on preferences, representing the value of the individuals’ health state. This instrument describes and evaluates health state in five dimensions: mobility, self-care, usual activities, pain/discomfort and anxiety/depression. Each dimension has three levels of severity: no problems (level 1), some problems (level 2) or extreme problems (level 3) lived or felt by the individual. The Portuguese version has a Cronbach’s alpha of 0.72 indicating a good internal consistency.^36^

### Ethical procedures

This study was approved by the Centro Hospitalar de Vila Nova de Gaia/ Espinho EPE’s Ethical Board (reference number 112-2018-1) in November 19, 2018. Signed informed consent was obtained after clear written and verbal explanation of the aim of the study and requirements for participants. Participants could drop out of the study at any point without fearing loss of quality of their care. All data were collected and managed following the General Data Protection Regulation (EU) 2016/679.

### Statistical analysis

Descriptive measures were calculated to describe our sample and their palliative care needs. Continuous measures were reported using the mean and standard deviation or median and range, according to their distributions. Distribution of continuous variables was assessed through visual inspection of the respective histogram.

Categorical variables were described using absolute numbers and proportions. To identify if there were any differences in characteristics and palliative care needs between individuals with and without an active DFU, we used Student’s *t*-test, for continuous variables with a normal distribution, or Mann–Whitney test, for those with asymmetric distribution. Finally, for the categorical variables, we used chi-square or Fisher’s exact test.

For all analysis, a *p*-value inferior to 0.05 was considered as statistically significant. The study methods and reporting were compliant with the STROBE checklist.^37^

## Results

### Sample characterization

We assessed the eligibility of 108 individuals and included 62 in our study (20 with and 42 without active DFU). The most common reasons for exclusion were active ulcer not caused by diabetes or not located in the foot (alone or concomitant with a DFU) (*n* = 22, 48%), hearing impairment (*n* = 5, 11%), inability to understand and respond to the questionnaires (*n* = 12, 26%) and refusal to participate (*n* = 7, 15%). The excluded participants had a mean age of 70 years, were mainly male (63%), with type 2 diabetes (94%) and with active DFU (52%), and by the end of the study, seven had died (15%).

In Table 1, we have described our sample’s demographic and clinical characteristics and compared those with and without an active DFU. In sum, the included sample had a mean age of 67 years, participants were mainly male (63%), with an education level up to or below the elementary school (63%), complete autonomy for activities of daily living (65%) and had type 2 diabetes (95%). Mean body mass index was 29 kg/m^2^, waist circumference 107 cm and HbA1c 7.6%. In total, 30 participants used insulin, 21 antidepressants and 7 were receiving medications for pain control. The majority had foot deformity (73%), neuropathy (66%) and history of previous DFU (53%).

**Table 1.** Sample characterization and differences between individuals with and without active DFU.

Variables	Total (*n* = 62)	With DFU (*n* = 20)	Without DFU (*n* = 42)	*p*
Demographic and clinical				
Age [*M* (*SD*)]	67 (10)	66 (8)	68 (10)	0.65^a^
Male sex [*n* (%)]	39 (63)	12 (60)	27 (64)	0.74^b^
Elementary education (4 years or less) [*n* (%)]	39 (63)	16 (80)	23 (55)	**0.09** ^b^
Physical autonomy [*n* (%)]	40 (65)	10 (50)	30 (71)	**0.09** ^b^
BMI (in kg/m^2^) [*M* (*SD*)]	29 (6)	31 (5)	29 (6)	**0.06** ^a^
Waist circumference [M (*SD*)]	107 (16)	112 (14)	104 (16)	**0.09** ^a^
DM related				
Type 2 DM [*n* (%)]	59 (95)	19 (95)	40 (95)	0.68^c^
DM duration [median (range)]	16 (1–47)	23 (3–44)	12 (1–47)	0.18^d^
Hypertension [*n* (%)]	46 (74)	15 (75)	31 (74)	1.0^b^
HbA1c (in %) [*M* (*SD*)]	7.6 (1.3)	7.8 (1.3)	7.5 (1.3)	0.51^a^
History of stroke [*n* (%)]	12 (19)	4 (20)	8 (19)	1.0^c^
History of myocardial infarction [*n* (%)]	8 (13)	5 (25)	3 (7)	**0.1** ^c^
Presence of retinopathy [*n* (%)]	28 (45)	10 (50)	18 (43)	0.6^a^
Presence of nephropathy [*n* (%)]	10 (16)	4 (20)	6 (14)	0.7^c^
Use of medication				
Insulin [*n* (%)]	30 (48)	11 (55)	19 (45)	0.5
Antidepressive [*n* (%)]	21 (34)	4 (20)	17 (41)	0.11^a^
Painkiller [*n* (%)]	7 (11)	5 (25)	2 (5)	0.03^c^
Foot related				
Presence of foot deformity [*n* (%)]	45 (73)	16 (80)	29 (69)	0.4^a^
Altered sensation to SWM or tuning fork [*n* (%)]	41 (66)	17 (85)	24 (57)	0.03^a^
Altered pulses palpation [*n* (%)]	23 (38)	11 (58)	12 (29)	0.03^a^
History of DFU [*n* (%)]	33 (53)	15 (75)	18 (43)	0.02^a^
History of LEA [*n* (%)]	15 (24)	8 (40)	7 (17)	**0.06** ^c^

In bold p-values equal or inferior to 0.1.

BMI, body mass index; DFU, diabetic foot ulcer; DM, diabetes mellitus; LEA, Lower Extremity Amputation; SD, standard deviation; SWM, Semmes-Weinstein monofilament.

a Student’s *t*-test.

b Chi-square test.

c Fisher’s exact test.

d Mann–Whitney test.

Those with an active DFU were significantly taking more often pain medication, had neuropathy, ischaemia and history of previous DFU (*p* < 0.05).

For those with active DFU, we have described the ulcer characteristics in Table 2. We can observe that the majority had a single ulcer (79%) that had a median duration of 3 months. The ulcers were more frequently located in the forefoot, deep and accompanied with ischaemia or neuropathy, but had less frequently bacterial infection or an area superior to 1 cm^2^. SINBAD median score was of 3, ranging from 0 to 5.

**Table 2.** DFU description using SINBAD classification (*n* = 20).

Variables	*n* (%)
Site (midfoot or hindfoot)	3 (15)
Ischaemia (presence)	11 (55)
Neuropathy (presence)	15 (75)
Bacterial infection (presence)	7 (35)
Area (more than 1 cm^2^)	5 (25)
Depth (reaching muscle, tendon or bone)	11 (55)

SINBAD, Site, Ischaemia, Neuropathy, Bacterial Infection and Depth.

### IPOS questionnaire

In Table 3, we describe the prevalence of each symptom assessed using the IPOS questionnaire and if there were differences between those with and without an active DFU. In Table 4, we report the severity of each symptom in the overall sample and in each group under analysis.

**Table 3.** IPOS questionnaire symptoms prevalence difference between participants with and without an active DFU.

Item	Total (*n* = 62)	With DFU (*n* = 20)	Without DFU (*n* = 42)	*p*
	*n* (%)	*n* (%)	*n* (%)	
Q2 – Physical symptoms				
Pain	34 (55)	11 (55)	23 (55)	1.0
Shortness of breath	16 (26)	1 (5)	15 (36)	0.01
Weakness or lack of energy	21 (34)	7 (35)	14 (33)	0.9
Nausea	3 (5)	2 (10)	1 (2)	0.2
Vomiting	2 (3)	0 (0)	2 (5)	1.0^a^
Poor appetite	10 (16)	5 (25)	5 (12)	0.2
Constipation	17 (27)	5 (25)	12 (29)	0.8
Sore or dry mouth	22 (36)	6 (30)	16 (38)	0.5
Drowsiness	33 (53)	11 (55)	22 (52)	0.8
Mobility	38 (61)	14 (70)	24 (57)	0.3
Q3 – Feeling anxious	42 (68)	19 (95)	23 (55)	0.002
Q4 – Family anxiety	60 (97)	20 (100)	40 (95)	0.3
Q5 – Feeling depressed	33 (53)	13 (65)	20 (48)	0.2
Q6 – Feeling at peace	24 (39)	8 (40)	16 (38)	0.9
Q7 – Sharing feelings	41 (66)	18 (90)	23 (55)	0.006
Q8 – Information	12 (19)	4 (20)	8 (19)	0.9
Q9 – Practical matters	30 (48)	12 (60)	18 (43)	0.2

DFU, diabetic foot ulcers; IPOS, Integrated Palliative care Outcome Scale.

a All *p* values were calculated using chi-square test, except for Vomiting (^a^) in which we used the Fisher’s exact test.

**Table 4.** IPOS questionnaire responses and differences between individuals with and without active DFU.

Item	Response [*n* (%)]	Total (*n* = 62)	With DFU (*n* = 20)	Without DFU (*n* = 42)
Q2 – Physical symptoms				
Pain	Not at all	28 (45)	9 (45)	19 (45)
	Slightly	16 (26)	5 (25)	11 (26)
	Moderately	8 (13)	3 (15)	5 (12)
	Severely	9 (15)	3 (15)	6 (14)
	Overwhelmingly	1 (2)	0 (0)	1 (2)
Shortness of breath	Not at all	46 (74)	19 (95)	27 (64)
	Slightly	9 (15)	1 (5)	8 (19)
	Moderately	5 (8)	0 (0)	5 (12)
	Severely	0 (0)	0 (0)	0 (0)
	Overwhelmingly	0 (0)	0 (0)	0 (0)
Weakness or lack of energy	Not at all	41 (66)	13 (65)	28 (67)
	Slightly	7 (11)	3 (15)	4 (10)
	Moderately	7 (11)	1 (5)	6 (14)
	Severely	7 (11)	3 (15)	4 (10)
	Overwhelmingly	0 (0)	0 (0)	0 (0)
Nausea	Not at all	59 (95)	18 (90)	41 (98)
	Slightly	1 (2)	1 (5)	0 (0)
	Moderately	1 (2)	0 (0)	1 (2)
	Severely	1 (2)	1 (5)	0 (0)
	Overwhelmingly	0 (0)	0 (0)	0 (0)
Vomiting	Not at all	60 (97)	20 (100)	40 (95)
	Slightly	1 (2)	0 (0)	1 (2)
	Moderately	1 (2)	0 (0)	1 (2)
	Severely	0 (0)	0 (0)	0 (0)
	Overwhelmingly	0 (0)	0 (0)	0 (0)
Poor appetite	Not at all	52 (84)	15 (75)	37 (88)
	Slightly	4 (7)	3 (15)	1 (2)
	Moderately	3 (5)	1 (5)	2 (5)
	Severely	3 (5)	1 (5)	2 (5)
	Overwhelmingly	0 (0)	0 (0)	0 (0)
Constipation	Not at all	45 (73)	15 (75)	30 (71)
	Slightly	7 (11)	1 (5)	6 (14)
	Moderately	4 (7)	1 (5)	3 (7)
	Severely	4 (7)	2 (10)	2 (5)
	Overwhelmingly	2 (3)	1 (5)	1 (2)
Sore or dry mouth	Not at all	40 (65)	14 (70)	26 (62)
	Slightly	9 (15)	3 (15)	6 (14)
	Moderately	8 (13)	2 (10)	6 (14)
	Severely	5 (8)	1 (5)	4 (10)
	Overwhelmingly	0 (0)	0 (0)	0 (0)
Drowsiness	Not at all	29 (47)	9 (45)	20 (48)
	Slightly	18 (29)	5 (25)	13 (31)
	Moderately	12 (19)	5 (25)	7 (17)
	Severely	3 (5)	1 (5)	2 (5)
	Overwhelmingly	0 (0)	0 (0)	0 (0)
Mobility	Not at all	24 (39)	6 (30)	18 (43)
	Slightly	11 (18)	3 (15)	8 (19)
	Moderately	15 (24)	7 (35)	8 (19)
	Severely	12 (19)	4 (20)	8 (19)
	Overwhelmingly	0 (0)	0 (0)	0 (0)
Q3 – Feeling anxious	Not at all	20 (32)	1 (5)	19 (45)
	Occasionally	4 (7)	3 (15)	1 (2)
	Sometimes	13 (21)	7 (35)	6 (14)
	Most of the time	19 (31)	8 (40)	11 (26)
	Always	6 (10)	1 (5)	5 (12)
Q4 – Family anxiety	Not at all	2 (3)	0 (0)	2 (5)
	Occasionally	6 (10)	1 (5)	5 (12)
	Sometimes	14 (23)	7 (35)	7 (17)
	Most of the time	27 (44)	9 (45)	18 (43)
	Always	12 (21)	3 (15)	10 (24)
Q5 – Feeling depressed	Not at all	29 (47)	7 (35)	22 (52)
	Occasionally	8 (13)	7 (35)	1 (2)
	Sometimes	15 (24)	3 (15)	12 (29)
	Most of the time	3 (5)	0 (0)	3 (7)
	Always	7 (11)	3 (15)	4 (10)
Q6 – Feeling at peace	Always	38 (61)	12 (60)	26 (62)
	Most of the time	7 (11)	3 (15)	4 (10)
	Sometimes	10 (16)	5 (25)	5 (12)
	Occasionally	5 (8)	0 (0)	5 (12)
	Not at all	2 (3)	0 (0)	2 (5)
Q7 – Sharing feelings	Always	21 (34)	2 (10)	19 (45)
	Most of the time	24 (39)	13 (65)	11 (26)
	Sometimes	9 (15)	3 (15)	6 (14)
	Occasionally	4 (7)	1 (5)	3 (7)
	Not at all	4 (7)	1 (5)	3 (7)
Q8 – Information	Always	50 (81)	16 (80)	34 (81)
	Most of the time	6 (10)	3 (15)	3 (7)
	Sometimes	5 (8)	1 (5)	4 (10)
	Occasionally	1 (2)	0 (0)	1 (2)
	Not at all	0 (0)	0 (0)	0 (0)
Q9 – Practical matters	Problems addressed/no problems	32 (52)	8 (40)	24 (57)
	Problems mostly addressed	22 (36)	9 (45)	13 (31)
	Problems partly addressed	0 (0)	0 (0)	0 (0)
	Problems hardly addressed	6 (10)	3 (15)	3 (7)
	Problems not addressed	2 (3)	0 (0)	2 (5)

DFU, diabetic foot ulcers; IPOS, Integrated Palliative care Outcome Scale.

A total of 27 participants reported one main problem or concern, 8 reported two and 2 participants reported three (Question 1). This first question of IPOS (‘What have been your main problems or concerns over the past week?’) is an open question, with the majority of the participants reporting issues related to their health, family and financial issues.

The most frequent symptoms (Question 2) were pain (55%), drowsiness (53%), weakness or lack of energy (44%) and sore or dry mouth (35%). Nausea and vomiting were described by less than 5% of our sample. Those without an active DFU significantly presented more shortness of breath. The remaining symptoms had similar distribution between both groups.

However, 1 patient reported two symptoms that were not addressed by IPOS and 19 patients reported one additional symptom. Each patient addressed a different symptom, such as stomach pain, problems with memory, numbness and others.

Most participants felt anxious or worried about their illness or treatment (Question 3) sometimes (*n* = 13) or most of the time (*n* = 19). Those with an active DFU reported feeling like this in 95% of the cases in comparison with 55% of the cases without (*p* = 0.002).

Most patients reported having family or friends anxious or worried with them sometimes (*n* = 14) or most of the time (*n* = 27) (Question 4). Most felt depressed (Question 5) occasionally (*n* = 8) or sometimes (*n* = 15), but seven individuals reported feeling like this always. Feeling at peace always (*n* = 38) or most of the time (*n* = 7) was reported by 72% of the individuals (Question 6).

Only 10% of the participants with an active DFU said that they were always able to share how they felt with family and friends as much as they wanted (Question 7) in comparison with 45% of those without an active DFU (*p* = 0.006).

In 91%, individuals reported having had as much information as they wanted (Question 8) and in 88% having their problems addressed (*n* = 32) or mostly addressed (*n* = 22) (Question 9) always or most of the time.

In Table 5, we present the IPOS subscales values. The subscales with higher values were emotional symptoms (41 ± 22), followed by communication/practical issues (23 ± 21) and physical symptoms (15 ± 14). There were no statistically significant differences between groups, although subjects with DFU had slightly higher values.

**Table 5.** Descriptive statistics and distribution for IPOS subscales scores.

Scale	Total (*n* = 62)		With DFU (*n* = 20)		Without DFU (*n* = 42)	
	*M* (*SD*)	Range	*M* (*SD*)	Range	*M* (*SD*)	Range
Physical symptoms	15 (14)	0–60	15 (13)	0–48	15 (14)	0–60
Emotional symptoms	41 (22)	0–100	43 (16)	0–81	40 (24)	0–100
Communication/practical issues	23 (21)	0–88	25 (17)	0–56	22 (22)	0–88

DFU, diabetic foot ulcers; IPOS, Integrated Palliative care Outcome Scale; M, mean; SD, standard deviation.

### EQ-5D-3L questionnaire

In Table 6, the answers to the EQ-5D-3L questionnaire are provided. Most patients reported having some problems with mobility (*n* = 42) but no problems with undertaking self-care (*n* = 38) or usual activities (*n* = 34). Almost half of the individuals described feeling pain/discomfort (*n* = 29) or anxiety/depression (*n* = 26) at moderate or extreme levels. No differences were found between patients with and without DFU.

**Table 6.** EQ-5D questionnaire responses and differences between individuals with and without active DFU.

Item	Response [*n* (%)]	Total (*n* = 62)	With DFU (*n* = 20)	Without DFU (*n* = 42)	*p* ^a^
Mobility	No problemsSome problemsUnable	19 (31)42 (68)1 (2)	4 (20)15 (75)1 (5)	15 (36)27 (64)0 (0)	0.2
Self-care	No problemsSome problemsUnable	38 (61)21 (34)3 (5)	11 (55)9 (45)0 (0)	27 (64)12 (29)3 (7)	0.5
Usual activities	No problemsSome problemsUnable	34 (55)26 (42)2 (3)	8 (40)11 (55)1 (5)	26 (62)15 (36)1 (2)	0.1
Pain/discomfort	NeitherModerateExtreme	33 (53)27 (44)2 (3)	10 (50)10 (50)0 (0)	23 (55)17 (40)2 (5)	0.7
Anxiety/depression	NeitherModeratelyExtremely	36 (58)24 (39)2 (3)	13 (65)7 (35)0 (0)	23 (55)17 (41)2 (5)	0.4

DFU, diabetic foot ulcers.

a Comparing lowest score answer with all remaining (e.g. no problems *versus* all others).

## Discussion

### Main findings

Our study shows that using a PCOM, it is possible to identify palliative needs in the population with or at risk of diabetic foot complications. Comparing with other palliative care studies, this population has a lower symptom prevalence and lower scale values.^25,34^ Despite all participants receiving continuous outpatient/ambulatory care and being mostly satisfied with the information provided and health problems addressed (91% and 88%, respectively), there are issues, especially in emotional dimensions of the patients’ life, not being followed up. This occurs probably because these symptoms are not often evaluated in these population due to time and staff restraints but also for these domains are not always considered as urgent, limb or life-threatening, which is the focus of care in the high-risk diabetic foot setting. Hence, we consider this identification as the first step to provide adequate palliative care provision earlier in the disease trajectory of these patients.

Surprisingly, physical symptoms had a similar frequency between those with and without an active DFU. Although there were differences in the use of medication for pain control between the groups, there were no statistically significant differences found in the item pain in IPOS or pain/discomfort in EQ-5D-3L. Most of the included participants reported pain independently of having or not an active DFU with a similar distribution in the prevalence (reported in 55% of participants in both groups) and severity of this symptom. This may be explained by a five times higher use of pain killers in those with an active DFU and by higher prevalence of peripheral neuropathy (85% *versus* 57%). However, our participants had a mean age of 67 years, with a mean BMI of 29 (considered as overweight) and a waist circumference of 107 cm. These characteristics are frequently, for example, linked with musculoskeletal degenerative and inflammatory processes.^38,39^

Drowsiness, constipation and sore or dry mouth were also frequently reported. These symptoms are consistent with the expected high prevalence of autonomic neuropathy in this tertiary setting.

The only significant difference found was that those without an active DFU presented more shortness of breath. This might be due to differences in underlying conditions or a result of the recommendation that patients with an active DFU reduce weight-bearing activities, which usually lead to less exertion.

We observed that those with active DFU seem to present more emotional and psychological symptoms and also a significantly lower ability to talk with family and friends about how they felt. In fact, only 1 in 10 of the patients with an active DFU reported being able to share their feelings as much as they wanted. In a recent qualitative study, Nielsen *et al.*^40^ found that when facing the possibility of amputation people in this situation does not tend to talk about it neither with their family or friends and specially not with strangers (the theme becomes a taboo, and they usually feel misunderstood). This is in line with our findings and highlights the importance of training healthcare professionals in communication to allow the creation of moments for these patients to inform how their disease is impacting their emotions^19^ or even the participation in support groups for these patients, as suggested by participants in the mentioned study.^40^

Chrisman,^12^ in her literature revision, resumed the most stressful events for people with chronic wounds: pain, exudate leakage, restricted mobility, poor hygiene, feelings of disgust or shame because of disfigurement or malodor, sleep disturbance, loss of sexuality, dissatisfaction with treatments, loss of control, social isolation, dependency, residency relocation, anger and lack of confidence in the healthcare provider because of failure to heal. Although there are questionnaires that perfectly address this stressors (e.g. Wound-QoL),^41^ that are useful for symptoms identification to improve patients’ conditions, they miss the articulation with palliative care treatment. IPOS scale may not focus on all the referred stressors, but it allows each individual to identify his most important symptoms (in both open and closed questions) and covers physical, social, psychological and spiritual domains. Our study showed that most of our patients in the diabetic foot context present pain, drowsiness, impaired mobility, anxiety, depression and that they are unable to share their feelings. This highlights the importance of social, psychological and spiritual support. However, only after the identification of these needs, will it be possible for those healthcare professionals to refer patients to specialized mental health consults, for further assessment. There is a severe lack of such professionals in diabetic foot teams all over the world. Our results support the need for a massive shift in this reality and we consider that this change could improve clinical outcomes, patients’ adhesion to treatment and overall well-being.^18^

### Strengths and weaknesses of the study

We believe this to be the first study identifying palliative care needs in patients at risk of diabetic foot complications and using such IPOS along with EQ-5D-3L to better understand individuals with or at risk of developing an active DFU. Although there are some recommendations about the palliative care needs of diabetic population,^42,43^ to our knowledge, there are no studies identifying those needs in people at risk of DFU.

Clinical and foot characterization variables were collected by a podiatrist with experience in diabetic foot and IPOS and EQ-5D-3L questionnaires were applied by a clinical psychologist in a quiet room improving the internal validity of data collection. However, this may affect the external validity of results. The researchers who developed IPOS scale, focused on creating a generalized use of the questionnaire, and concluded that with training, any health professional could use the scale.^26^

We have conducted our study in only one setting; hence, results may not be generalizable. On the one hand, we acknowledge that conducting the study at a tertiary care institution may overestimate the palliative care needs. On the other hand, this context is the one that makes more clinical sense and in which multidisciplinary diabetic foot teams exist.

Although it may be somewhat time-consuming, the IPOS questionnaire, with training, is considered to be easy to apply and helps in providing adequate treatment to these patients.^25^ This reinforces the necessity of sensitizing health professionals caring for diabetic foot patients to include this tool in their clinical care^44^ and Hospital managers to include palliative care specialists in the teams that treat patients at high risk of diabetic foot complications.

One of the major limitations is the low sample size of our study. Despite the effort of including all eligible patients followed in the clinic for 9 months, the sample was still low (62 participants), specially for comparing the group with (*n* = 20) and without (*n* = 42) DFU. Although we have used a consecutive inclusion sampling technique, during the period of inclusion, we have excluded 46 patients, presenting several of them difficulty in hearing, understanding or responding to the questionnaire.

Patients who were excluded tended to be older and with more severe DFUs. We highlight that, by the end of the study, 15% of the patients excluded had died in comparison with 0% of the included. This suggests that we may have a selection bias leading to an underestimation of palliative care needs. Therefore, in future studies, we consider that a multicentre study including a larger number of people with different characteristics should be conducted as it would not only increase the precision of the findings but also their generalizability. We also consider that less strict selection criteria could be applied (for example, to include people with other type of active ulcers besides diabetes-related or with hearing or communication impairment). Nevertheless, a subgroup analysis should be made to understand if there are differences in the results between them.

### What this study adds

Our study suggests that people with high risk or with active diabetic foot complications present a high level of pain, emotional and psychological distress. Those individuals with active DFU tend to report more palliative care needs but also less ability to communicate their feelings. In health systems, one difficulty is to promptly identify DFUs and refer for multidisciplinary specialist foot care (these referrals are frequently delayed or absent).^45^ The other is to include palliative care in diabetic foot care or at least to establish effective protocols with palliative care specialists.^20^

Further multicentre studies with large sample sizes are needed to characterize this population more substantially and to understand the impact of adequate palliative care in clinical results and on patients’ well-being.

## Declarations

**Ethics approval and consent to participate:** This study was approved by the Centro Hospitalar de Vila Nova de Gaia/ Espinho EPE’s Ethical Board (reference number 112-2018-1) in 19 November 2018. Signed informed consent was obtained after clear written and verbal explanation of the aim of the study and requirements for participants. Participants could drop out of the study at any point without fearing loss of quality of their care. All data were collected and managed following the General Data Protection Regulation (EU) 2016/679.

**Consent for publication:** Not applicable.

**Availability of data and materials:** Access to the data was only granted in line with that consent, subject to approval by the project ethics board. Supporting data are available on request: please contact Matilde Monteiro-Soares through matsoares@med.up.pt.
